# Understanding Child-Directed Speech Around Book Reading in Toddler Classrooms: Evidence From Early Head Start Programs

**DOI:** 10.3389/fpsyg.2021.719783

**Published:** 2021-12-09

**Authors:** Annemarie H. Hindman, Jean M. Farrow, Kate Anderson, Barbara A. Wasik, Patricia A. Snyder

**Affiliations:** ^1^Department of Teaching and Learning, Temple University, Philadelphia, PA, United States; ^2^Graduate School of Education, University of Pennsylvania, Pennsylvania, PA, United States; ^3^Center for Social Organization of Schools, Johns Hopkins University, Baltimore, MD, United States; ^4^Anita Zucker Center for Excellence in Early Childhood Studies, University of Florida, Gainesville, FL, United States

**Keywords:** toddler, child-directed speech (CDS), early childhood education, teacher, book reading

## Abstract

Child-directed speech (CDS), which can help children learn new words, has been rigorously studied among infants and parents in home settings. Yet, far less is known about the CDS that teachers use in classrooms with toddlers and children’s responses, an important question because many toddlers, particularly in high-need communities, attend group-care settings. This exploratory study examines the linguistic environment during teacher-led book readings in American Early Head Start classrooms serving 2-year-olds from households in poverty. Seven teachers in four classrooms were trained to emphasize target words while reading story and informational books. We first analyzed the nature and quality of their book readings from a macro-level, exploring global instructional quality [Classroom Assessment Scoring System (CLASS)] and linguistic complexity [i.e., diversity of vocabulary (*D*) and sophistication of syntax (MLU-w)], and we also examined micro-level teacher-child talk strategies and use of target words. Compared to prior research, these classrooms had similar global quality and syntactic complexity, although less lexical diversity. Exploratory results also revealed three distinct teacher talk patterns—teachers who emphasized (1) comments, (2) questions, and (3) a balance of the two. Question-focused teachers had more adult and child talk during reading, as well as more repetitions of target words, and stronger CLASS Engaged Support for Learning. However, comment-focused teachers used more diverse vocabulary and had stronger CLASS Emotional and Behavioral Support. Results illuminate the nature and quality of CDS in toddler classrooms, particularly in the context of an intervention emphasizing target vocabulary words, and highlight applications for professional development and questions for further research.

## Vocabulary Development

One of the most important milestones of the first years of life is learning language, beginning with vocabulary (Samuelsson, 2021). Knowing more words in early childhood facilitates further vocabulary and language development, a virtuous cycle (Peter et al., 2020; Avila-Varela et al., 2021). Children with more vocabulary knowledge have, both immediately and over time, greater success in reading and other content areas (Dickinson et al., 2010; Cristofaro and Tamis-LeMonda, 2012; Morgan et al., 2015), better-adjusted social interactions, and more self-regulation and executive functioning (Winsler, 2009; Manning et al., 2019; Rantalainen et al., 2021). Unfortunately, growing up in poverty is, as early as 18 months of age, associated with less knowledge of vocabulary and slower language processing (Fernald et al., 2013; Suggate et al., 2018), making a focus on these children’s early experiences a priority.

## Child-Directed Speech

Beyond considerable individual differences in how and how quickly children learn words (Fernald et al., 2006; Donnelly and Kidd, 2020), the language input children receive from those around them plays a key role in their language learning (Abend et al., 2017; Golinkoff et al., 2019; Fitch et al., 2020). When communicating with very young children, adults often systematically alter how they talk, using specialized, child-directed speech (CDS) that draws children’s attention and highlights the sounds in words, supporting vocabulary and other language outcomes (Bryant and Barrett, 2007; Zauche et al., 2016). Most often studied among pre-verbal infants, CDS is characterized by unusual auditory features such as high pitch, slow pace, exaggerated prosody, and distinct timbre; as well as sparse word volume and frequent repetition of words, focus on concrete ideas, and simple syntactic structure (Rowe, 2008, 2012; Huttenlocher et al., 2010; Longobardi et al., 2016; Quick et al., 2019; Genovese et al., 2020; Rowe and Snow, 2020). Adults’ CDS changes as children progress into toddlerhood (e.g., 1–2 years of age) and begin to talk and respond on their own (Durán et al., 2004; Hoff, 2014) using one-, two-, or three-word phrases (i.e., telegraphic speech) (Rice et al., 2010) undergirded by basic syntax and grammar (Hoff et al., 2018; Cadime et al., 2019). Adults’ CDS to toddlers employs more standard prosody and longer utterances, with more numerous and complex words and grammatical structures, as well as increased back-and forth through extended adult-child conversations fostered by questions (Rowe, 2012; Longobardi et al., 2016).

However, despite all we know about CDS, most research targets parents and families, with far less work examining interactions between teachers and young children, especially toddlers. In many American communities, particularly those in poverty, approx. 60% of toddlers attend care settings, with 12% in center-based classrooms and 30% in home-based group care (Paschall, 2019). Unfortunately, the quality of teachers’ CDS many be low (LaParo et al., 2009), demanding further research and professional development (PD).

## Child-Directed Speech in Early Childhood Classrooms

To date, the literature on CDS in toddler classrooms is a patchwork, with a variety of different ways of defining, measuring, and aggregating components of teacher CDS and a mix of observational and PD intervention studies. A critical review of this literature reveals three relatively distinct approaches to conceptualizing/measuring CDS: global conceptual quality, linguistic complexity, and specific teacher (and, occasionally, child) talk strategies. Below, we review key findings from each approach, focusing on toddlers but, because of the small literature, including data from preschool when relevant and necessary.

### Global Quality

Global quality measures assign one score to an entire instructional activity block or day. CDS lies at the heart of global quality scores, but other features of teaching (e.g., materials) and child activities factor in as well. Building on the classic tools such as the Early Childhood Environment Rating Scale (ECERS) and the Early Language and Literacy Classroom Observation (ELLCO), considerable recent research has supported the reliability and validity of the Classroom Assessment Scoring System (CLASS), which can be used in classrooms from infancy through high school (Hamre et al., 2012, 2013). The CLASS-Toddler includes two domains, each with several dimensions: Emotional and Behavioral Support (Positive Climate, Negative Climate, Teacher Sensitivity, Regard for Child Perspectives, Behavior Guidance) and Engaged Support for Learning (Facilitation of Learning and Development, Quality of Feedback, and Language Modeling). At present, we are beginning to understand global quality in toddler settings. National samples in United States toddler classrooms suggest that CDS related to Emotional support is typically high (*M* = 5.30, *SD* = 0.07, on a scale from 1 to 7) while Engaged Support for Learning is modest (*M* = 3.60, *SD* = 0.15) (Bandel et al., 2014). Because CLASS-T scores have been predictive of child vocabulary and language outcomes in prior work (e.g., Aikens et al., 2015), we include CLASS-T in this study.

### Linguistic Complexity

Other approaches have focused on the overall linguistic complexity of teachers’ CDS in an instructional activity block or day.

#### Lexical Diversity

Lexical diversity, or the percentage of unique (rather than repeated) words used, has typically been measured using a type-token ratio (i.e., the number of distinct, different words and their inflections and derivations relative to the number of total words). Because CDS features simplified word choice, expanding as children age, studies have generally found values around 0.17 in toddlers’ households (Hoff, 2003; Rowe, 2012), meaning that only 17% of the words children hear are words they heard only once during the language sample; most words were repeated multiple times. Interestingly, Girolametto et al. (2003) explored toddler and preschool classroom CDS and found a ratio of 0.44 during book reading, far higher than home settings. More recent work, however (Montag et al., 2018), has found that type-token ratio should be adjusted to account for the length of the language sample, resulting in widespread adoption of a novel measure of lexical diversity referred to as *D*. The construct of *D* has been used in preschool research; for example, Dickinson et al. (2014) found that teacher CDS in Head Start book readings averaged 74.41, aligned with a type/token ratio of 0.58. It is helpful to note that average *D* for children’s talk at age 2 is 27.44 and the average at age 3 is 47.83 (Durán et al., 2004). To our knowledge, *D* for teachers’ CDS has not been widely gauged in toddler classroom settings, which we explore in the current study.

#### Structural Complexity

Another feature of CDS is simple language construction, such as “That’s a dog!” (for infants) to “That’s a big, furry dog, and it’s running down the street” (for toddlers). Complexity can be measured with mean length of utterance (MLU-w), or number of words per statement. In preschool book readings, Dickinson et al. (2014) found an average MLU-w of 8.39, indicating that teachers generally used about 8 words per remark. Less research is available on toddler classrooms, but Girolametto et al. (2003) reported teacher MLU-m (a slightly more liberal measure than MLU-w) around 5.03 during book reading. It is helpful to note that toddlers’ average MLU-w is 2.90 (Girolametto and Weitzman, 2002). The current study consequently explores teachers’ MLU-w.

More specific still, Justice et al. (2013) examined more nuanced features of the syntax of CDS, adapting Huttenlocher et al. (2010)’s approach to capture constructions that boost MLU-w, including simpler strategies (e.g., verb phrases, prepositional phrases, adjectives/adverbs) and more complex strategies (subordinate clauses, modifying verbal phrases, advanced phrases, compound sentences). They found that, among preschoolers, these constructions are generally used infrequently, but to our knowledge, this study is the first to code toddler teachers’ CDS In this way.

### Specific Talk Strategies

A third, highly nuanced approach to understanding CDS is a micro-level examination of teachers’ specific words, including the teachers’ language modeling (including their conceptual complexity and their use of specific vocabulary words), inviting child talk (including questions that promote conversational turns), and feedback. Relatedly, a few studies have explored child talk, both in response to and independent of teacher talk.

### Teacher Language Modeling

Abundant evidence suggests that an important aspect of teachers’ CDS is modeling language through their remarks, which has been understood from two primary perspectives. First, as laid out in Dickinson and Tabors (2001), the *conceptual complexity of teachers’ talk* matters, specifically whether it is contextualized (i.e., in reference to information that is immediately apparent, such as on the page of a book, including labeling or describing an illustration) or decontextualized (i.e., in reference to abstract information, such as a synthesis or prediction). Both teacher and/or parent contextualized and decontextualized talk in book reading and other conversational settings supports children’s vocabulary outcomes (Pancsofar and Vernon-Feagans, 2006; Rowe, 2012), but decontextualized talk may be particularly supportive of learning for children with stronger language skills (Pellegrini et al., 1990; Reese, 1995; Currenton et al., 2008; Hindman et al., 2008). Book reading studies in preschool have shown that more complex books offer exposure to more complex language via the text and engender more decontextualized talk (Dickinson et al., 2014; Muhinyi et al., 2020).

Another aspect of teacher CDS is *teachers’ use of specific vocabulary words*. The language development literature indicates that frequent exposure to language aids in learning it (Ambrose et al., 2015), and one extension is that multiple repetitions of the same vocabulary words, ideally with rich interactions around them, can support children in learning them (Harris et al., 2011; Beck et al., 2013). Adult talk about and repetition of specific target words learn can build child knowledge of those words (Kaiser and Trent, 2007; McLeod et al., 2017), and at least one study with preschoolers (Wasik and Hindman, 2020) suggests that children’s standardized receptive and expressive vocabulary skills increase when teachers use target words more frequently. Interestingly, toddler teachers may tend to repeat target words more often than preschool teachers, perhaps to match children’s emerging language skills (Girolametto and Weitzman, 2002; Girolametto et al., 2003). This target word focus may have powerful results—one Early Head Start study (Romano and Woods, 2018) trained three teachers in several types of strategies including modeling target phrases (see Roberts et al., 2014), which was ultimately linked to gains in children’s talk, including target words. In this study, we track teacher language modeling (complexity) and frequency of target word use.

#### Teacher Questions/Back-and-Forth

Beyond what teachers say lies the degree to which teachers invite child talk and foster extended exchanges. *Teacher questions* often start conversations (Rowe et al., 2017; Gilkerson et al., 2018). Questions can be of many formats (Wasik and Hindman, 2013), including open (involving no single correct answer and likely requiring multiple word response) or closed (single correct answer), with the latter including label questions (What’s this called?) or yes/no questions (Is this a dog?). There is extensive evidence from preschool that questions in the classroom are a powerful tool for eliciting child language and fostering conversation (Whitehurst, 2004). Open-ended questions may be relatively rare, representing approx. 5% of preschool teachers’ prompts (e.g., Siraj-Blatchford and Manni, 2008), but closed questions can help to check understanding (Wasik and Hindman, 2013).

Among toddlers, however, there is little research, but some evidence suggests that patterns of teacher questions are similar. For example, Davis et al. (2015) examined six classrooms serving children ages birth to two and determined that teachers predominately asked the same kinds of questions seen in preschools—closed yes/no questions and closed label questions, with relatively few open-ended questions. Interestingly, O’Brien and Bi (1995) found that toddlers often did not respond to teacher questions, perhaps because their language skills were not yet strong enough. Similarly, Kidd and Rowland (2021) found that, with 2-year-olds as well as 3-year-olds, when presented with conversational opportunities, children contributed just about one-third (37%) of the turns. They hypothesized that more teacher-dominated conversations (such as emphasizing closed questions) with younger children may be supportive of language. In the current study, we track teachers’ questions (open/closed).

#### Teacher Responsiveness/Feedback

Beyond modeling language and then inviting child talk, research has increasingly focused on a third component of teacher-child exchanges—unpacking how and how much (e.g., conversational turns) teachers *respond to child talk*. Much research has examined the value of teachers’ extending children’s language (for example, responding to “Dog!” with “That is a dog!”) and/or elaborating on children’s ideas (for example, responding to “Dog!” with “That dog is brown and furry”), both of which are linked to important gains in a variety of children’s language skills (Cabell et al., 2015). Relatively less research (Cabell et al., 2015; Casla et al., 2021) has targeted other contingent teacher remarks; for example, Kidd and Rowland (2021) also highlighted ignoring, copying/repeating, rephrasing, and interpreting, finding some likely benefits of these talk strategies as well. To our knowledge, teachers’ feedback to children has not been widely studied in toddler classrooms, and we examine this question in the current study, including this variety of feedback strategies.

#### Child Talk

Even though CDS changes as children begin to talk (Rowe and Snow, 2020), it has often been conceptualized as something of a one-way street, with the content and meaning of the exchange exclusively driven by the adult and with less attention to child talk (Golinkoff et al., 2015). Justice et al. (2018) established, at least implicitly, the critical importance of child talk in their discovery that the most important driver of child language in preschool classrooms was teacher invitations for child talk. In one relevant study that included child talk, Wasik and Hindman (2011) offered PD to 19 Head Start teachers and, during teacher-led book readings, coded both *children’s responses to teachers’ questions* (distinguishing between single- and multiple-word responses) and *children’s spontaneous talk.* Findings showed that children’s vocabulary growth was uniquely linked to more child talk during book reading. A subsequent study (Champagne, 2019) investigated the *accuracy* of preschoolers’ responses and teachers’ feedback on errors, finding that teachers tended to call on children whom they presumed would have the correct answer, and that incorrect responses were rare. We build on this work to explore toddlers’ responses and spontaneous talk, including accuracy, with a particular focus on discussion of target words.

## Interrelations and Patterns

Thus, across the literature, teacher CDS has been explored from a collection of macro- and micro-level perspectives. But to date, just two studies have explored these aspects of CDS (and child language) simultaneously; examining how they co-occur and whether there may be “styles” or “registers” has key implications for PD. First, Justice et al. (2018) examined how three ways of capturing CDS—global quality, linguistic complexity, and several kinds of talk strategies (encouraging child language)—uniquely predicted vocabulary in 49 preschool classrooms when considered together. Results showed that overall, these different facets of CDS were uncorrelated with one another, but that only teacher talk strategies (specifically, encouraging child talk) predicted vocabulary learning. Second, Dickinson et al. (2014) examined linguistic complexity and several kinds of talk strategies (modeling language, discussing literacy, social studies, etc.). As in Justice et al. (2018), these different facets of CDS were relatively independent of one another. However, book reading specifically offered evidence of an academic language register or style, wherein more vocabulary focus, greater lexical diversity, and greater structural complexity clustered together. The current study is, to our knowledge, the first to explore the correlations and patterns among the multiple facets of CDS in toddler classrooms, including a wide array of teacher and child talk strategies, to understand their overlap and independence.

## Current Study

In sum, teacher CDS has been examined through varied lenses, including global instructional quality, linguistic complexity, and specific talk strategies, but there is great variability in methods and results across studies, and very little work targeting high-need toddler contexts. Because the toddler period is essential for language and vocabulary growth, and because high-quality teacher CDS can foster this growth, we developed and piloted a PD intervention for Early Head Start teachers. *Head Start on Vocabulary* (HSoV) is built on an effective preschool teacher PD model (Wasik et al., 2006; Wasik and Hindman, 2011, 2020) involving training teachers in language modeling, questioning, and feedback, all focused on target words, during book reading. In this study, we explored how toddler teachers used CDS during book reading after *HSoV* training and supports, as well as how children used language, particularly target words.

### Research Questions

We explored several research questions in a small sample of 7 teachers that enabled close examination of classroom CDS:

First, what is the *global quality* of *HSoV* teachers’ classroom instruction and CDS? We used a gold-standard measure, the Classroom Assessment Scoring System—Toddler tool.

Second, what is the *linguistic complexity*, including lexical diversity and structural complexity, of teachers’ CDS during book reading? We used the well-established CHILDES and CHAT language coding approach.

Third, what is the nature of *teachers’ and children’s talk strategies* during book reading? We considered several elements of the adult-child exchange: teachers’ language modeling, children’s responses, and teachers’ responses to children. We also explored the nature of child-initiated talk during book reading, and teachers’ responses. We examined both central trends and individual differences or patterns across teachers.

Fourth, how frequently are *target words* mentioned in the text, by teachers, and by children during book reading? We employed simple frequency counts.

Finally, to what extent are these *three lenses on CDS correlated or unique* from one another? We employed Pearson zero-order correlations.

Together, these questions explore teachers’ CDS and child talk around vocabulary in high-need toddler settings, in the context of the target-word-focused *HSoV* intervention.

## Materials and Methods

### Procedure

In fall 2019, we partnered with a local Early Head Start provider in a major urban city in the American northeast to develop *Head Start on Vocabulary (HSoV)*. Program administrators identified a total of four toddler (ages 2–3 year) classrooms from two centers. Each classroom was team-taught by two co-teachers, and all eight consented to participate.

#### Head Start on Vocabulary

To our knowledge, there are no widely available interventions to support toddler teachers’ CDS with rigorous evidence of effectiveness. In preschool, however, several effective interventions have improved the quality of preschool teachers’ talk to children, resulting in gains in preschoolers’ knowledge of taught words as well in standardized vocabulary scores (Landry et al., 2011; Weiland and Yoshikawa, 2013), including our own model which we have developed and tested over 20 years (Wasik and Bond, 2001; Wasik et al., 2006; Wasik and Hindman, 2011, 2020). In this project, we adapted our preschool program to address center-based toddler teachers’ classroom CDS, developing *HSoV* for Early Head Start.

Like our preschool model, *HSoV* supported toddler teachers over a full academic year, including several distinct components that, taken together, offer a relatively “light touch.” First, we offered *group workshops.* Beginning in fall, teachers attended 90-min monthly group workshops for 4 months. Workshops addressed (1) Talking to children using descriptive language, especially regarding new vocabulary; (2) Asking children questions, particularly about new vocabulary; (3) Assisting children in answering questions, if needed; and (4) Providing feedback on what children have said, emphasizing new vocabulary. Each began with an interactive lecture by project staff, presenting the rationale for and explanation of the target strategy and inviting teachers to share their experiences and concerns. We also demonstrated the strategies and shared short (1–3 min) videos of teachers in urban centers using the strategies with fidelity. Finally, we explained the classroom materials teachers would receive (see below) and gave teaching teams time to plan with support from project staff, so they could prepare to use the strategies on their own.

To support the use of the four strategies through book readings and play-based extension activities, we provided one trade book per week, to be read at daily to each child, either in groups or one-on-one. From each book, we selected three words that likely to be unfamiliar to children (Beck et al., 2013). For example, for the book *Little Blue Truck Leads the Way* (Schertle and McElmurry, 2015), we selected the words truck, road, and traffic jam. We then created extension activities for four different classroom areas (e.g., housekeeping, construction) to reinforce the words (e.g., teachers might read and then visit the construction area to act out a traffic jam together). Finally, we provided an 8.5″× 11″ full-color picture card of each target word, with a child-friendly definition on the back, for the teacher share with children daily.

After each workshop, teachers received *coaching* every other week, including a direct 30-min observation in their classroom by a master teacher and who videotaped the teacher engaging in various activities, including book reading. The coach then watched the videos, took notes, and offered feedback in 45-min one-on-one on-site conferences.

Observations continued until March 2020, at which point the classrooms closed because of COVID-19. This study makes use of the videos collected by the coach in late fall 2019-winter 2020, after teachers had been through the training and were using the strategies in their classrooms. All study procedures were conducted with the approval of our university’s IRB.

### Participants

A total of seven teachers participated in the project, as the 8th left the classroom before video collection began, and we did not record the substitutes who temporarily replaced her. All teachers were women of African-American backgrounds. All were native speakers of English. All held a minimum of a high school degree, while two were working toward an associate’s degree and two others held an associate’s degree. Although this study focuses on teachers, each classroom served 8 children (teacher: child ratio of 1:4), all between 24 and 36 months of age. The sample was evenly divided by gender. All children were of backgrounds that are minoritized in the U.S., with African American (60%), Hispanic-Latino (30%), and/or Asian (10%) heritage. Approximately 50% of children spoke a home language other than English (primarily Spanish).

### Measures

Table 1 summarizes measures and key variables in the current study.

**TABLE 1:** Variables in the current study.

Construct	Measure	Variables in current study	Possible range of values
**Global quality**			

	CLASS-Toddler	Emotional and behavioral support Positive climate Recoded negative climate Teacher sensitivity Regard for child perspectives	All items measured on a 1–7 scale; Sub-domains and domains represent the average of all relevant items, so will be scored from 1 to 7.
		Engaged support for learning Facilitation of learning and development Quality of feedback Language modeling	

**Linguistic complexity**			

	CHAT and CLAN standardized coding schemes	Linguistic diversity: *D:* Total word types/total words, adjusted for length of language sample	Minimum = 0, Maximum = 1
		Structural complexity: Mean length of utterance—w: Average number of words per utterance	Minimum = 0, Maximum unbounded
	Justice et al. (2013) coding scheme	Inclusion of specific syntactic constructions Simpler constructions: verb phrases, prepositional phrases, adjectives/adverbs More complex constructions: subordinate clauses, modifying verbal phrases, advanced phrases, compound sentences	Frequency counts Minimum = 0, Maximum unbounded

**Talk strategies**			

	Project-derived coding schemes	Teacher remarks Child responses Teacher responses to child responses Child-initiated Talk Teacher responses to child-initiated talk target words	Frequency counts Minimum = 0, Maximum unbounded

#### Teacher Background

A background survey collected contact data and demographic information (e.g., education, ethnicity). The paper-pencil survey required about 5 min.

#### Overall Classroom Quality

We used the Classroom Assessment Scoring System—Toddler (CLASS-T) version (La Paro et al., 2011), targeting two domains, each with several sub-domains: Engaged Support for Learning (i.e., Facilitation of learning and development, Quality of feedback, and Language modeling), as well as Emotional and Behavioral Support (i.e., Positive climate, Negative climate, Teacher sensitivity, Regard for child perspectives, and Behavior guidance). Widely used and considered a gold-standard tool, the CLASS-T has strong psychometric properties, including construct validity and inter-rater reliability (Hamre et al., 2012). A trained rater watched videos and then scored the classroom instruction on a variety of items (all rated 1-very low quality to 7-very high quality), yielding an average for each domain and sub-domain. Because the CLASS is most reliable when more minutes of instruction are coded, we coded all parts of every teacher’s video. On average, videos (including but not limited to the book reading segment) coded with CLASS-T were 10.47 min long (*SD* = 2.96, *range* = 4.93–13.58).

#### Linguistic Complexity of Teachers’ Child-Directed Speech

Videos of book readings were transcribed for analysis in the Codes for Human Analysis of Transcripts (CHAT) from the Child Language Data Exchange System (CHILDES), with analyses of syntactic complexity (MLU-w) and lexical diversity (*D*) of teacher talk conducted using Child Language Analysis (CLAN) program (MacWhinney, 2000). Transcriptions began as soon as the teacher indicated the activity was starting (e.g., “Are all my friends ready to look at our new book?”) and ended when the teacher announced the conclusion (e.g., “Okay, you all did a good job today”). The average length of the book reading portion of the videos was 6.00 min (*SD* = 3.07, *range* = 2.42–13.38).

#### Transcription

Teachers’ speech was parsed into C-units (Loban, 1976), utterances defined as containing one main clause and any modifying phrases and subordinating clauses. Thus, the following example – “The boy is jiggling his ears/and he’s shaking his leg” – would be parsed into two separate C-units. We did not code the syntax of teachers’ reading of actual text from the book, as the purpose was to analyze the complexity of teachers’ CDS. All transcripts were checked twice, in addition to using automated check features within the CLAN program before analysis. An example is provided in **Supplementary Appendix**.

#### Lexical Diversity

Teachers’ quantity of input was calculated using the FREQ command. CLAN derived the total amount of words and word types within the transcripts of teachers’ lessons. We used D as our measure of lexical diversity (McKee et al., 2000), because unlike type-token ratios, D accounts for differences in length of language samples, allowing for comparisons across transcripts, and thus more accurately measuring lexical diversity. The VOCD command in CLAN calculated D for teachers.

#### Structural Complexity

We used MLU-w as a proxy for complexity of speech, in that longer word utterances often include words, phrases, and clauses that modify meaning of the main clause (Hoff, 2003). As with lexical diversity, CLAN derived the MLU-w of teachers using the MLU command. We further coded teachers’ use of complex language structures in their CDS using a scheme from Justice et al. (2013). We captured relatively simpler strategies (e.g., verb phrases, prepositional phrases, adjectives/adverbs) and more complex strategies (subordinate clauses, modifying verbal phrases, advanced phrases, compound sentences). We calculated the frequency of each kind of structure, as well as the proportion of teachers’ total utterances that included one or more of these structures.

#### Talk Strategies in Child-Directed Speech

We coded every teacher utterance during the book readings to understand how teachers were using specific conversational strategies in their CDS. Our coding scheme followed previous work in the field (e.g., van Kleeck et al., 2003; Hindman et al., 2008; Wasik and Hindman, 2020) but included new codes as needed. We distinguished among (A) Teacher-initiated talk, (B) Child responses to teacher-initiated talk, (C) Child-initiated talk, and (D) Teacher responses to children. We also coded each sentence that teachers read from the book as (E) Reading Text to track the number of sentences per book. We coded directly from video, without transcription. We coded every video twice to ensure accuracy. An example is provided in **Supplementary Appendix**.

##### Defining Utterances

As with the syntax coding, we began coding when teachers announced the start of the activity and ended when teachers moved on to a different activity. Also aligned with the syntax coding, we generally defined an utterance as an independent clause, or a remark that included, at a minimum, a subject and verb. Therefore, a sentence such as, “What’s he doing right here?” would be one utterance, whereas “He’s dancing and his friend is laughing” would be two utterances. However, children generally did not speak in complete sentences, so stand-alone remarks such as “Red!” (meaning, “That thing is red”) were also coded as utterances. Finally, we used the same logic in coding brief, stand-alone teacher responses to child remarks, allowing one-word responses such as “Right!” to be coded.

##### Teacher-Initiated Talk

We coded every teacher-initiated remark, focusing on content and format. Regarding content, we coded remarks as either *contextualized* (related to information apparent on the page of the book) or *decontextualized* (involving abstraction or inference) (Dickinson and Tabors, 2001). For example, questions about illustrations (e.g., “What color is her shirt?”) were contextualized, but references to real-life experiences, summaries, or predictions (e.g., “What do you think Max will do next?”) were decontextualized.

Regarding format, we coded every teacher-initiated remark as a comment, an open prompt, or a closed prompt. *Comments* were statements that did not request a child response (e.g., “There’s the cat”). Types of comments included labeling/describing, defining a word, or providing other information. *Open prompts* were those in which more than one correct answer was possible (e.g., “What do you see on this page?”). *Closed prompts* were those in which the correct answer was limited to one option. We coded for several types of closed questions, including (a) label-related (e.g., “What color is this cat?”), (b) choice questions (e.g., “Is this a boat or a car?”), (c) yes/no questions (e.g., “Is she sitting down in this picture?”), (d) point or gesture questions (e.g., “Point to the car”), and (e) requests to repeat (e.g., “Say ‘car”’). Notably, prompts included remarks that were technically statements but that functioned as questions (e.g., “Tell me what you see here”).

##### Child Responses to Teacher-Initiated Talk

We tracked how children responded to each teacher open and closed prompt. We found that content and format of child responses were determined by the initial teacher remark, so within each category of prompt [open, closed (label, choice, yes/no, gesture, repeat)], we focused on whether the child response was correct, incorrect, or no response. We considered both verbal responses and gestures, noting the latter.

We did not track the identities of individual children, and we did not distinguish between responses provided by one child vs. those provided simultaneously by several children (given the quality of our audio and the varied group size within and across videos). Accordingly, when a teacher posed a question to the group and only one child responded (a frequent pattern), we coded only the speaking child’s response (correct/incorrect) and excluded the other children’s non-response. In addition, we observed one a situation where two children offered a response at the same time, one of which was correct while the other was incorrect. In this case, we marked the response as correct (and the teacher responded to the correct response). Overall, then, our coding scheme privileged correct child responses, and estimates of child talk can be viewed as describing the top end of the possible frequency and accuracy distribution.

##### Child-Initiated Talk

We coded spontaneous child remarks without any prompt from the teacher. In the absence of extensive prior research, we adapted our child response codes. We coded for spontaneously *repeating* what a teacher just said; labeling a *target word* (“Boat!”); labeling something that was not a target word (“Dog!” as the child notices a dog in a picture); offering a *description* of a picture, often related to color or (“Red!” when looking at a picture of Elmo or “Ribbit!” when looking at a picture of a frog); and offering a question about a picture (“What he doing?”). We intended to code both child responses and spontaneous remarks as contextualized or decontextualized, but all child-initiated remarks were contextualized.

##### Teacher Responses to Child Talk

Finally, we coded teachers’ responses to children’s talk, based on prior approaches. Codes included *repeating* the child verbatim (“Boat!”), repeating the child and *adding words* (“That’s a boat!”), *adding a new idea* (“A boat can sails on the water”) and *praising* the child (“Great job!”). We also included, for teacher responses to child incorrect remarks, asking a *rhetorical question* (“You sure that’s a car?”), *re-asking* the same question, and *rephrasing/reframing* a question as choice or yes/no. Finally, We coded for giving a *hint* (“Like a car, but it drives on special tracks….”) and for giving the *correct answer*.

#### Target Word Frequency

In a final round of coding, we tallied the number of times that target words—vocabulary related to the text and/or theme—were used. We separately counted for target words (1) in the text itself, as well as (2) in teacher talk, and (3) in child or children’s talk. We counted each instance of use of a target word, even when repetitive; for example, if a teacher said, “That’s a tire—there’s the tire,” we counted both mentions of the target word *tire*. In addition, we counted an individual child using the target word or all children simultaneously using the target word as one instance. Ultimately, we had three values for each classroom—total mentions of target words in the text, teacher talk, and child talks.

## Results

### Question 1: Global Quality of Classroom Interactions

Complete results are presented in Table 2. On average, teachers in this sample demonstrated levels of CLASS-T Emotional and Behavioral Support in the moderate/high range (*M* = 5.00, *SD* = 0.90, *range* = 3.4–6.4). Within this first domain, the highest average dimension score was on (Recoded) Negative Climate (*M* = 6.88, *SD* = 0.33, *range* = 6.0–7.0), indicating very little harshness or negativity. The lowest average score was on Behavior Guidance (*M* = 3.70, *SD* = 1.40, *range* = 1.0–6.0), indicating that more effective guidance could be provided in many classrooms.

**TABLE 2:** Descriptive statistics for overall classroom quality (CLASS-T).

CLASS-T domain	M	SD	Range
Emotional and behavioral support	5.00	0.90	3.40–6.40
Positive climate	5.45	1.27	3.00–7.00
Recoded negative climate	6.88	0.33	6.00–7.00
Teacher sensitivity	4.97	1.01	3.00–6.00
Regard for child perspectives	3.90	1.72	2.00–7.00
Behavior guidance	3.69	1.40	1.00–5.00
Engaged support for learning	3.81	1.43	1.30–6.00
Facilitation of learning and development	4.24	1.46	2.00–6.50
Quality of feedback	3.18	1.59	1.00–6.00
Language modeling	3.97	1.47	1.00–6.00

On average, the domain of Engaged Support for Learning in these classrooms also fell into the moderate range (*M* = 3.81, *SD* = 1.43, *range* = 1.3–6.0). The dimension with the highest value was Facilitation of Learning and Development (*M* = 4.24, *SD* = 1.46, range = 2.0–6.5), and the lowest was on Quality of Feedback (*M* = 3.18, *SD* = 1.59, *range* = 1.0–6.0). We examined Pearson zero-order correlations to explore the degree to which the dimensions and domains were correlated; domains were moderately to highly correlated with one another (*r* = 0.60, *p* = 0.10), and dimensions were generally correlated with the domain to which they pertain.

### Question 2: Linguistic Complexity of Teachers’ Language

See Table 3 for complete results. Teachers read an array of different books, including *The Bus for Us* (Bloom, 2001), *Baby Loves Winter* (Katz, 2013), *Froggy Gets Dressed* (London and Remkiewicz, 1994), *Time for a Bath* (Gershator and Walker, 2014), *Ready Set Brush* (Rudko, 2008), and *Shake a Leg!* (Allen and Swanson, 2010). All books were provided by the *Head Start on Vocabulary* intervention and were similar in length and complexity. On average, teachers used 75.86 total utterances (*SD* = 26.17, range = 44–106) with children during their book readings, comprised of an average of 547.14 words (*SD* = 229.26). However, the range was wide, with the teacher at the lowest end of the distribution using as few as 317 words while the teacher at the highest end used 944 words, approx. 300% as many words.

**TABLE 3:** Descriptive statistics of linguistic complexity of teachers’ talk during book reading.

Language feature	Mean	SD	Min	Max
**CHAT coding**				

Total utterances	75.86	26.17	44.00	106.00
Mean length of utterances-words	6.14	0.73	5.11	7.06
Total words	547.14	229.26	317.00	944.00
Total types	105.14	11.61	87.00	119.00
Type/token ratio	0.22	0.07	0.12	0.32
Lexical diversity (D)	32.06	7.35	19.62	39.50

**Proportion of utterances with complex syntactic components**				

Complex utterances	0.20	0.09	0.11	0.34
Simple utterances	0.80	0.09	0.66	0.89
Embedded clause	0.14	0.07	0.09	0.28
Verbal phrase	0.06	0.05	0.02	0.17
Advanced phrase	0.01	0.01	0.00	0.04
Verb phrase	0.36	0.09	0.27	0.54
Prepositional phrases	0.21	0.08	0.12	0.33
Compound structures	0.05	0.03	0.01	0.09
Adjective or adverb	0.15	0.10	0.05	0.34

#### Lexical Diversity

Children heard an average of 105.14 (*SD* = 11.61, range = 87–119) different word types; framed another way, teachers used many of the same words over and over again). Type/token ratio was, on average, 0.22 (*SD* = 0.07, *range* = 0.12–0.32). Teachers’ lexical diversity (*D*) mean scores, which account for the number of words in the sample (McKee et al., 2000), averaged 32.06 (*SD* = 7.35, range = 19.62–39.50).

#### Structural Complexity

Regarding MLU-w, each utterance, on average, contained 6.14 words (*SD* = 0.73, *range* = 5.11–7.06). It is helpful to note that children’s utterances at age 2 generally involve 1–3 words, meaning that teachers’ language was more complex than children’s, with twice as many words per utterance, on average. Pearson correlations showed that teachers’ lexical diversity (*D*) was not related to syntactic complexity (MLU-w), (*r* = 0.05, *p* = 0.908).

Results of additional syntax coding (see Justice et al., 2013) revealed that teachers extended the length of their utterances with simpler constructions. Teachers used verb phrases (e.g., He will go home) in 36% of utterances, prepositional phrases l that specify and describe a noun or event (e.g., “The snow is falling *from the sky*”) in 21% of utterances, and phrases with and adjectives or adverbs (e.g., “The truck picks up the *stinky* trash) in 15% of utterances. They also used more complex syntactic structures, including embedded or subordinating clauses (e.g., “Can we pay the driver *so that we can ride our taxi*?”) in 15% of utterances, verbal phrases (e.g., “Do you see how happy Froggy is *playing outside with snow coming down*?”) in 6% of remarks, and compound structures (e.g., “Froggy wants to go play, *but he has no socks*!”) in 5% of utterances, and advanced phrases (e.g., “The children build the base, *the bottom*, of the snowman”) in 1% of remarks. It is helpful to note that 2–3 year old children’s language rarely includes either these more or less complex syntactic augmentations (Vasilyeva et al., 2008), making teachers’ syntax more complex than children’s.

### Question 3: Teacher and Child Talk Strategies

Below, we present results in order of their role in the teacher-child exchange: (1) Teacher-initiated talk, (2) Child responses to teacher talk, and (3) Teacher responses to child responses. We also include (4) Child-initiated talk not in response to a teacher remark, and (5) Teacher responses to child-initiated talk. Results are presented in Table 4.

**TABLE 4:** Descriptive statistics of talk moves.

Talk move	Mean	*SD*	Min	Max
**Teacher remarks**				

All teacher-initiated remarks	48.86	12.40	32.00	67.00
Teacher comments	21.71	11.76	8.00	46.00
Teacher comment—label	19.14	10.51	5.00	40.00
Teacher comment—define	0.00	0.00	0.00	0.00
Teacher comment—decontextualized	2.43	1.90	0.00	5.00
Teacher open-ended questions	1.00	1.52	0.00	4.00
Teacher open-ended contextualized	0.57	1.51	0.00	4.00
Teacher open-ended decontextualized	0.43	0.79	0.00	2.00
Teacher closed questions	15.28	11.02	4.00	34.00
Teacher closed—label	3.57	4.12	0.00	11.00
Teacher closed question—choice	1.86	4.49	0.00	12.00
Teacher closed question—yes/no	5.71	6.63	0.00	17.00
Teacher closed question—repeat	2.43	3.15	0.00	9.00
Teacher closed question—action	0.71	1.89	0.00	5.00
Teacher closed question—point	1.00	2.65	0.00	7.00
Teacher closed question—decontextualized	0.14	0.38	0.00	2.00

**Child responses**				

All child responses	16.57	10.94	5.00	35.00
All child correct responses	8.71	8.26	2.00	22.00
All child incorrect/no responses	7.86	3.89	2.00	13.00
Point—correct	0.71	1.89	0.00	5.00
Point—incorrect	0.14	0.38	0.00	1.00
Point—no response	0.14	0.38	0.00	1.00
Repeat—correct	0.86	0.90	0.00	2.00
Repeat—incorrect	0.00	0.00	0.00	0.00
Repeat—no response	1.71	2.98	0.00	8.00
Choice—correct	1.29	3.40	0.00	9.00
Choice—incorrect	0.57	1.13	0.00	3.00
Choice—no response	0.00	0.00	0.00	0.00
Yes/no—correct	4.28	5.59	0.00	14.00
Yes/no—incorrect	0.71	1.11	0.00	3.00
Yes/no—no response	0.71	1.25	0.00	3.00
Word—correct	0.86	1.57	0.00	4.00
Word—incorrect	1.28	1.25	0.00	3.00
Word—no response	1.57	1.81	0.00	4.00
Open-ended—correct	0.14	0.38	0.00	1.00
Open-ended—incorrect	0.00	0.00	0.00	0.00
Open-ended—no response	0.86	1.21	0.00	3.00
Gesture—correct	0.57	1.51	0.00	4.00
Gesture—incorrect	0.00	0.00	0.00	0.00
Gesture—no response	0.14	0.38	0.00	6.00

**Teacher responses, child incorrect**				

No response	3.14	3.44	0.00	10.00
Repeat child	0.86	1.07	0.00	3.00
Rhetorical question	0.86	1.21	0.00	3.00
Re-ask same question	1.57	1.72	0.00	4.00
Reframe as choice question	0.57	1.51	0.00	4.00
Reframe as Yes/No question	0.57	0.98	0.00	2.00
Give hint	1.14	1.68	0.00	4.00
Explicit no	0.14	0.38	0.00	1.00
Give correct answer	2.14	1.68	0.00	5.00
Ask child to repeat their answer	0.43	0.79	0.00	2.00
Ask follow-up question	0.28	0.75	0.00	2.00

**Teacher responses, child correct**				

No response	0.85	1.21	0.00	3.00
Repeat child label	1.14	1.77	0.00	5.00
Rhetorical question	0.14	0.38	0.00	1.00
Repeat child other	1.00	2.24	0.00	6.00
Praise	2.43	4.03	0.00	11.00
Explicit yes	0.57	0.79	0.00	2.00
Add words to child response	1.71	2.50	0.00	7.00
Expand child idea	1.43	1.62	0.00	4.00
Ask follow-up question	0.00	0.00	0.00	0.00
Child-Initiated Talk	5.00	3.79	1.00	9.00
All child-initiated talk	7.00	6.16	2.00	20.00
Voluntarily repeats teacher	1.71	2.21	0.00	5.00
Volunteers target word	1.14	1.46	0.00	4.00
Volunteers description of picture	0.43	0.79	0.00	2.00
Volunteers other information	1.28	0.75	0.00	2.00
Volunteers question	0.014	0.38	0.00	1.00
Teacher response, child-initiated talk				
All teacher response to child-initiated talk	7.00	6.16	2.00	20.00
No response	1.28	2.21	0.00	5.00
Repeat child	1.14	1.07	0.00	3.00
Rhetorical question	0.28	0.38	0.00	1.00
Praise	0.00	0.00	0.00	0.00
Explicit no	0.00	0.00	0.00	0.00
Explicit yes	2.28	2.21	1.00	7.00
Add words to child response	1.57	2.23	0.00	6.00
Expand child idea	1.14	1.35	0.00	4.00
Ask follow-up question	0.57	0.79	0.00	2.00

#### Teacher-Initiated Talk

Teachers initiated 48.86 total book-related utterances during their book readings (*SD* = 12.40, range = 32–67). Of these, comments predominated (*M* = 21.71, *SD* = 11.76, range = 8–46), representing on average 45% of total teacher talk (but the proportion ranged from 15 to 69% across teachers). Closed prompts were less frequent (*M* = 15.28, *SD* = 11.02, range = 4–34, accounting for 31% of teacher-initiated talk). Open-ended prompts were rare (*M* = 1.00, *SD* = 1.53, range = 0–4, 2%). Attention- and management-related remarks not directly related to the book itself accounted for 9.57 remarks (*SD* = 5.62, *range* = 4–18), or 19% of overall talk (*range* from 10 to 30%). Praise was offered on average once per reading (*M* = 1.14, *SD* = 1.46, *range* = 0–4), representing 3% of teacher talk (*range* = 0–8%).

##### Teacher Comments

The vast majority of teacher comments (about 20 per reading) were contextualized, primarily labeling/describing pictures (*M* = 19.14, *SD* = 10.51, *range* = 5–40). Decontextualized remarks occurred just twice per reading, on average (*M* = 2.43, *SD* = 1.90, *range* = 0–5). There were no instances of teachers defining words. There were no correlations among types of comments; teachers who used one type did not necessarily use other types.

##### Teacher Closed Prompts

The vast majority of closed prompts targeted contextualized information, with yes/no questions related to labeling (e.g., “Is this a cat?) used most frequently (*M* = 5.71, *SD* = 6.63, *range* = 0–17). Asking children to provide a label (e.g., “What’s this?”) was also relatively common (*M* = 3.57, *SD* = 4.12, *range* = 0–11), as was choosing between two labels (*M* = 1.86, *SD* = 4.48, *range* = 0–12) and repeating a label (*M* = 2.43, 3.15, *range* = 0–9). Many additional strategies were used by only one teacher; for example, the teacher in video #4 used a single decontextualized closed question (“Where do boats drive?”), the teacher in video #16 asked children point to pictures of a target word on seven occasions, and the teacher in video #15 asked children to act out target words five times. Only one correlation among closed prompts was observed: there was a high correlation (*r* = 0.82, *p* = 0.02) between asking for labels and asking choice questions.

##### Teacher Open-Ended Prompts

Open-ended prompts were used less than once/reading, on average (*M* = 0.57, *SD* = 1.51, *range* = 0–40 and *M* = 0.43, *SD* = 0.79, *range* = 0–2, respectively). Only one teacher (video #6) used a contextualized open question (e.g., “What do you see on the cover of the book?”) but did so four times. Two teachers used decontextualized open questions (e.g., “What do you think could happen next?”), one (video #17) just once and the other (video #9) twice. We did not explore correlations, given their infrequency.

##### Patterns in Teacher-Initiated Talk

To explore patterns in teacher talk, we conducted zero-order Pearson correlations among these variables. Comments and open prompts (*r* = −0.19, *p* = 0.968) were independent of one another, as were open and closed prompts (*r* = −0.22, *p* = 0.639). However, there was an inverse, marginally significant correlation between comments and closed prompts (*r* = 0.74, *p* = 0.057), indicating that teachers who made more comments asked fewer closed questions. When closed and open prompts were combined (i.e., all prompts), there was significant inverse correlation between comments and questions (*r* = −0.76, *p* = 0.048). Thus, teachers in this sample appeared to either favor questions or comments in their book readings.

Descriptive data identified two teachers who used predominately questions rather than comments (41% of their talk was comments whereas 26% was questions and 64% was comments whereas 15% was questions, respectively), whom we termed *Asker* teachers. Conversely, four teachers used predominately comments rather than questions (43% comments to 29% questions, 63% comments to 25% questions, 59% comments to 15% questions, and 69% comments to 6% questions); we termed these teachers *Tellers*. One teacher, termed *Balanced*, used the two types of remarks about equally (44% comments to 38% questions). Descriptive data showed that Teller teachers used more comments (*M* = 26.75, *SD* = 13.00 vs. 12.00, *SD* = 5.66) while Asker teachers used more questions (*M* = 30.00, *SD* = 5.66 vs. *M* = 8.50, *SD* = 3.41). The Balanced teacher fell in between on comments (*n* = 21) and questions (*n* = 20). Exploratory, underpowered ANOVA results comparing Askers and Tellers showed that, while the frequency of comments was not statistically different (*p* = 0.216), Asker teachers employed significantly more questions, *F*(1, 4) = 36.80, *p* = 0.004. In an additional exploratory analysis, we examined whether Askers and Tellers differed in the kinds (rather than just the amount) of comments and questions they made. There were no differences for comments, but Askers used significantly more labeling questions (*M* = 8.50 vs. *M* = 0.75 for Tellers), *F*(1, 4) = 16.664, *p* = 0.015. As these exploratory findings support the distinction between these groups, we employ these categories as we explore the data below.

##### Summary

Teacher-initiated CDS during toddler book readings predominately focused on contextualized (labeling, describing) information. Among comments, labeling/describing remarks predominated, and among questions, yes/no and labeling questions were most frequent. Three patterns of teacher talk during book reading emerged: Askers, Tellers, and Balanced.

#### Child Responses to Teacher Prompts

Complete results are presented in Table 5 and summarized below.

**TABLE 5:** Correlations among constructs.

Constructs	2	3	4	5	6	7	8	9
1. CLASS-T Emotional and behavioral support	0.60*	0.14	0.65	0.14	−0.61	−0.63	0.02	
2. CLASS-T Engaged support for learning	1	1.57	0.20	−0.31	0.12	0.11	−0.01	−0.01
3. Book reading MLU-w		1	0.05	0.02	0.09	0.09	−0.32	0.15
4. Book reading *D*			1	0.00	−0.45	−0.45	−0.15	−0.32
5. All teacher comments				1	−0.76*	−0.74∼	0.52	−0.39
6. All teacher questions					1	0.99***	−0.20	0.70∼
7. All child responses						1	−0.23	0.69∼
8. All child-initiated talk							1	−0.02
9. Book reading length								1

∼p < 0.10 *p < 0.05, ^***^p < 0.001.

##### Child Response Frequency

We observed an average of 16.57 (*SD* = 10.94, *range* = 5–35) child responses to teacher remarks, closely aligned with the number of teacher questions. Beyond this sample average, however, there were differences between Asker and Teller classrooms, in that Asker teachers (*n* = 2) saw 30.5 child responses in their classrooms (*SD* = 6.36), while Teller teachers saw just 8.75 (*SD* = 3.30) child responses. Differences were significant in an (underpowered) ANOVA, *F*(1, 4) = 34.44, *p* = 0.004.

##### Child Response Accuracy

Overall, across the sample as a whole, children were correct 48% of the time and incorrect or non-responsive 52% of the time, but accuracy varied by question type. Approx. 5 responses per reading (*M* = 5.20) were to yes/no questions, and most were correct (75%). Of the approx. 4 responses to label questions per reading (*M* = 3.71), 23% were correct, 34% were incorrect, and 42% involved non-response. Responses to repeating questions (i.e., “Say ‘truck”’) were offered 2–3 times per reading (*M* = 2.57), and children were correct 33% of the time but did not respond 66% of the time. Responses to choice questions were offered nearly twice per reading (*M* = 1.85), with mostly (69%) correct responses.

##### Patterns

Interestingly, when comparing Asker and Teller classrooms, children in Asker classrooms offered significantly more correct answers (*M* = 20.00 and *SD* = 2.83 vs. *M* = 2.75 and *SD* = 0.95), *F*(1, 5) = 147.63, *p* < 0.001, but statistically equivalent numbers of incorrect answers (*M* = 10.50 and *SD* = 3.53 vs. *M* = 6.00 and *SD* = 3.91), *F*(1, 4) = 16.64, *p* = 0.015. Descriptive statistics showed that labeling questions in particular (“What is this?”) were substantially more prominent in Asker (*M* = 8.50) classrooms than Teller classrooms (*M* = 0.75).

##### Summary

Children responded correctly to about half of teacher questions. Choice and yes/no questions generally resulted in correct responses, while answers to labeling and request for repetition questions were most frequently incorrect.

#### Teacher Responses to Child Responses

We coded teacher responses to incorrect vs. correct child answers separately for clarity.

##### Addressing Incorrect Child Responses

When children were incorrect, the most common teacher response was non-response (*M* = 3.14, *SD* = 3.44, *range* = 0–10), used at least once by 6 out of 7 teachers, and quite often (10 times) by one teacher (#5). It was also quite rare to tell the child that the answer was not correct (only 1 teacher used this approach, and only once).

Most teachers also used one or more strategies aimed at leading children toward the answer, including re-asking the same question (*M* = 1.57, *SD* = 1.72, *range* = 0–4), used by 5 out of 7 teachers, or giving hints (3 out of 7, *M* = 1.14, *SD* = 1.68, *range* = 0–4). Less common (on average, less than once per reading) were asking the child a rhetorical question (“Are you sure that’s a dog?”), used by 3 teachers, or reframing the original question as a yes/no (2 teachers) or choice question (1 teacher). Quite rare was asking a follow-up question (1 teacher, used twice).

Ultimately, most teachers (6 out of 7) also offered the correct answer, on average this twice per reading (*M* = 2.14, *SD* = 1.68, *range* = 0–5).

##### Addressing Correct Child Responses

When children were correct, teachers used a different array of responses. Most common was praise (*M* = 2.43, *SD* = 4.03, *range* = 0–11), used by 4 of 7 teachers, one very frequently (11 times). In addition, five teachers repeated what children said verbatim (*M* = 2.14, *SD* = 2.67, *range* = 0–6), four teachers added words to what children said (*M* = 1.71, *SD* = 2.50, *range* = 0–7), and four expanded on their idea (*M* = 1.43, *SD* = 1.62, *range* = 0–4). Rarer strategies (used less than once per reading) included non-response (used once but by three teachers), asking rhetorical questions (used by one teacher, once), explicitly saying yes (used by three teachers), and asking follow-up questions (never used).

##### Patterns

Not surprisingly, because they posed more questions and accordingly had more child responses, Askers offered significantly more responses to children than Tellers did, *F*(1, 4) = 11.49, *p* = 0.028. Given the relatively small cell sizes for each type of teacher response, we did not examine further differences in specific responses between Askers and Tellers.

#### Child-Initiated Talk

On average 5 times per reading, children volunteered information (*SD* = 3.78, *range* = 1–9). Unlike child responses, child-initiated talk was nearly identical regardless of whether teachers were Tellers (*M* = 5.25, *SD* = 4.35) or Askers (*M* = 5.50, *SD* = 4.95), with no significant differences between groups, *F*(1, 4) = 0.01, *p* = 0.952. In light of small cell sizes, we did not examine group differences further and instead describe the whole sample.

Looking closely at child-initiated talk, with two exceptions (the same child, in one classroom), all volunteered information was accurate. This talk mostly (at least once per classroom) involved children sharing the color or label for an illustration (*M* = 1.71, *SD* = 1.11, *range* = 1–4). In four classrooms, children specifically volunteered a particular target word (*M* = 1.14, *SD* = 1.46, *range* = 0–4). In three classrooms, one child voluntarily repeated what teachers said (*M* = 1.71, *range* = 0–5); moreover, children who repeated the teacher did so more than once (from 3 to 5 times). Only once across all videos did a child volunteer a question.

Zero-order correlations showed that the frequency of child responses in classrooms was unrelated to the frequency of child-initiated talk (*r* = −0.23, *p* = 0.627).

##### Summary

In all classrooms, whether Teller or Asker settings, at least one child volunteered information, generally labeling/describing illustrations or repeating; nearly all was accurate. Child-initiated talk was less frequent than responses to teachers.

#### Teacher Responses to Child-Initiated Talk

While, as above, the most frequent teacher feedback strategy on child responses to teacher questions was non-response, teacher response to child-initiated talk was different. In fact, only two teachers ever used non-response to child-initiated talk; one did so four times and the other five times (*M* = 1.29, *SD* = 2.21, *range* = 0–5). The most common approach, used by all teachers at least once, was to explicitly say “yes” in response to a child-initiated remark; on average, this happened twice per reading (with one offering this feedback 7 times (*M* = 2.29, *SD* = 2.21, *range* = 1–7). Five teachers repeated children’s remarks, on average once per reading (*M* = 1.14, *SD* = 1.60, *range* = 0–3), while four added words to what children said, on average once per reading (*M* = 1.52, *SD* = 2.22, *range* = 0–6) and five expanded children’s ideas, on average once per reading (*M* = 1.14, *SD* = 1.34, *range* = 0–4). Three teachers posed at least one follow-up question (*M* = 0.57, *SD* = 0.79, *range* = 0–2).

##### Patterns

No clear patterns were apparent. Tellers gave slightly more frequent feedback on child-initiated talk (*M* = 8.50, *SD* = 7.77 and *M* = 5.50, *SD* = 4.95, respectively), but this difference was not significant, *F*(1, 4) = 0.23, *p* = 0.654.

##### Summary

Children volunteered information equally often, whether or not teachers emphasized asking questions. Teachers offered feedback (often a “yes”) on this child talk.

### Question 5: Frequency of Target Words in Teacher and Child Talk

Frequency counts of use of target words in text, teacher talk (whether initiations or responses) and child talk (whether initiations or responses) indicated that target words were very common in the text and related conversations. Texts included 24.28 target words (*SD* = 15.12) but ranged from 12 to 55. Teachers used, on average, 48.57 target words (*SD* = 26.87), but there was substantial variation (*range* = 22–101). On average, 78% of teacher remarks (whether initiated or responses) included a target word. Children used 8.71 target words (*SD* = 6.05, *range* = 0–15), so on average, 40% of child remarks (whether initiated or responses) contained a target word.

Correlations among these three variables showed that the number of target words in the text was unrelated to use of target words by teachers (*r* = 0.03, *p* = 0.952) or children (*r* = 0.08, *p* = 0.869). However, when teachers used more target words, children did as well (*r* = 0.76, *p* = 0.047).

#### Patterns

Although Askers and Tellers read texts with very similar numbers of target words (*M* = approx. 20 for both groups), Askers used target words about twice as often (*M* = 76.50, *SD* = 34.65) as did Tellers (*M* = 36.25, *SD* = 17.52), and children in Asker classrooms used target words about three times as frequently (*M* = 14.50, *SD* = 0.71, vs. *M* = 5.50, *SD* = 6.03). Differences were not statistically significant (*p* > 0.20).

#### Summary

Target words were prominent in teacher extra-textual talk, and to a lesser degree in child talk; they were particularly salient in Asker classrooms.

### Question 6: Global Quality, Linguistic Complexity, and Talk Strategies Correlations

Results are presented in Table 5. In general, these constructs were independent of one another. Linguistic complexity was unrelated to frequency of teacher or child talk (*r* = 0.15–0.35, *p* > 0.50). Global quality was independent of linguistic complexity (*r* = 0.30–0.60, *p* > 0.10) and frequency of teacher and child talk (*r* = 0.20–0.60, *p* > 0.15). Interestingly, correlations remained largely non-significant even when exploring individual kinds of talk and very specific, highly related CLASS-T dimensions (e.g., teacher responses to child remarks on our specialized coding scheme and CLASS-T teacher feedback), with four exceptions. First, linguistic complexity was marginally correlated with Positive Climate (*r* = 0.71, *p* = 0.069) and Behavior Guidance (*r* = 0.70, *p* = 0.083); in other words, more diverse teacher vocabulary was linked to stronger support for children’s emotional and behavioral well-being. Second, more teacher questions were linked to a less positive climate (*r* = −0.81, *p* = 0.027), as were more child responses (*r* = −0.082, *p* = 0.025); similarly, a marginally significant correlation emerged teacher questions and Recoded Negative Climate (*r* = −0.71, *p* = 0.076). Thus, all three aspects of CDS in this sample were relatively distinct across the sample as a whole, although there were some indications that aspects of Emotional and Behavioral Support were enhanced by more vocabulary-rich teacher talk and less child talk.

Analyses of this collection of talk strategies again found differences between Askers and Tellers. In Askers’ classrooms, teachers initiated and responded more overall (*M* = 84.50, *SD* = 14.85), relative to Tellers (*M* = 50.00, *SD* = 19.68), a marginally significant difference, *F*(1, 4) = 4.59, *p* = 0.099, with the Balanced classroom falling in between (65.00). Children in Asker classrooms also talked more (initiations and responses) (*M* = 36.00, *SD* = 1.41)—twice as much, on average–than those in Teller classrooms (*M* = 14.00, *SD* = 3.74), a significant difference, *F*(1, 4) = 58.67, *p* = 0.002, with the Balanced classroom falling in between (23.00).

Examining global quality and linguistic complexity, Tellers and Askers had very similar structural complexity (MLU-w) to their language (*M* = 6.11, *SD* = 0.76 vs. *M* = 6.21, *SD* = 1.20.18, respectively). However, echoing the correlations between dimensions of CLASS and CDS, Tellers had higher scores on CLASS Emotional and Behavioral Support domain (*M* = 5.05, *SD* = 0.85 vs. *M* = 4.00, *SD* = 0.57), while Askers had higher scores on the CLASS Engaged Support for Learning scale (*M* = 4.70, *SD* = 1.41, vs. *M* = 3.65, *SD* = 1.58). ANOVAs did not find significance for comparisons. Tellers also used more diverse vocabulary (*D*, *M* = 36.75, *SD* = 3.50 vs. *M* = 28.90, *SD* = 4.96), a marginally significant difference (*p* = 0.085).

#### Summary

Although these three perspectives on CDS were largely distinct (uncorrelated), Askers’ instruction trended toward more alignment with the CLASS teaching-oriented scale, whereas Tellers’ use of predominately teacher talk and more different words trended toward higher levels of emotional and behavioral support.

## Discussion

This exploratory, observational study teased apart multiple elements of CDS in the context of toddler classrooms, examining trends and individual differences. Teachers in this study received training from the *Head Start on Vocabulary* (HSoV) model, and we explored the classroom language environment (global quality, linguistic complexity, and teachers’ and children’s talk strategies) during book reading, a potentially vocabulary-rich part of the early childhood classroom.

Regarding general trends, we found that global classroom quality (CLASS-T) was moderate and generally equivalent to larger Early Head Start samples (Vogel et al., 2015: Emotional and Behavioral Support = 5.3, Engaged Support for Learning = 3.5) and to Head Start preschools (U.S. Department of Health and Human Services [DHHS], 2021: Emotional Support = 6.03, Classroom Organization = 5.78, Instructional Support = 2.94). During book reading, structural complexity (MLU-w) was approximately equal to preschool book reading values (Dickinson et al., 2014) and slightly higher than previous toddler book reading values (Girolametto et al., 2003), with teachers’ CDS averaging about 6 words per remark, and about 20% of remarks employing one or more syntactically complex constructions. During book reading, teachers talked about three times as often as toddlers did, and most child talk was in response to teacher talk. Teachers predominately labeled and described illustrations and asked closed questions that invited children to provide a specific label. About half of children’s responses were correct. Throughout this talk, target words were frequently used, especially by teachers (on average, in 78% of remarks). In a related finding, lexical diversity (*D*), however, was about half of what has been observed in book readings in preschool (Dickinson et al., 2014) or toddler classrooms (Girolametto et al., 2003), perhaps in part because of the *HSoV* focus on a small set of target words.

Beyond overall trends, three general patterns of CDS during toddler book readings emerged: Teller classrooms (*n* = 4) in which teachers used predominately comments, Asker classrooms (*n* = 2) in which they used predominately questions, and Balanced classrooms (*n* = 1) with an equal mix of both. In Asker classrooms, children talked more overall (frequently with correct answers), and both teachers and children used target words more frequently. Asker classrooms also had (descriptively, although not statistically) higher scores on the CLASS-T Engaged Support for Learning scale, while Teller classrooms performed better on the Emotional and Behavioral Support scale. Teller teachers also used (marginally) more diverse vocabulary. Findings reveal new information about CDS during book reading toddler classrooms, at least in the context of this target-word-focused *HSoV* intervention, and potential patterns of talk have implications future research in PD with this population of teachers.

### Head Start on Vocabulary Teachers’ Child-Directed Speech Emphasized Target Words

The field has not determined optimal teacher lexical diversity and syntactic complexity for 2-year-olds. On one hand, there is clear evidence from the field that exposure to an extensive selection of vocabulary, including abstract words, is predictive of longer term benefits (Hart and Risley, 1995; Dickinson and Tabors, 2001), as is exposure to complex syntax (Hoff, 2003). However, in these classrooms, consistent with *HSoV* aims, teachers repeatedly labeled concrete items/images (e.g., truck, toothbrush), likely limiting lexical diversity but increasing repetitions of focal words and in some cases, allowing for moderately sophisticated descriptions of pictures in the texts.

Interestingly, when teachers asked closed, contextualized questions (mostly labeling), children were incorrect in half of their responses. This result is particularly intriguing given that the words selected for *HSoV* were relatively simple and concrete. This finding may indicate that a high degree of focused repetition, with few distractors, is very appropriate for 2-year-old children in high-need settings. Indeed, this focused talk could, over time, help children build target word knowledge, which in turn would offer a foundation for more sophisticated understandings and more extensive contributions to conversations. However, if both complex and simpler language inputs matter, future research might carefully track the appropriate balance of the two, perhaps through tracing teachers’ introduction of new words and children’s adoption of those words to better understand the key pathways by which new words become salient for and familiar to children.

### Children Have Different Experiences With Tellers vs. Askers

A major finding from this work involved preliminary evidence that some teachers, at least during book readings with toddlers, emphasize questions (Askers) while others emphasize comments (Tellers), and yet others have a Balance. There were some clear advantages for children of Asker classrooms—teachers and children talked substantially more, particularly about target words, linked at least marginally to higher global quality around teaching. Although we do not have data on child language learning, children in Asker teacher classrooms tended to answer questions correctly, even though Asker teachers used more labeling questions, which were, overall, one of the more challenging kinds of questions.

On the other hand, there were positive aspects of Teller classrooms, in that teachers used more diverse vocabulary, which was linked to a more Positive Climate and to higher Behavior Guidance. At the same time, teacher questions and child responses, both of which were less frequent in Teller classrooms, were inversely correlated with CLASS-T Positive Climate. Future research on this point is needed, but it may be the case that more teacher-managed discussion supports a smoothly run classroom and/or showcases teachers’ support for children in ways that the CLASS-T is particularly sensitive to notice.

As only one classroom fell into the Balanced category, conclusions about this group are limited. However, a general implication of these broader findings is that there are potentially benefits of various patterns for children, and that teachers could potentially be trained to use different sets of strategies together. In addition, if these patterns or styles are widespread among teachers, coaches may need to gauge how teachers approach CDS during book reading and tailor PD to optimize that approach and include other talk strategies as well.

## Limitations and Future Directions

A number of limitations to the current work highlight future directions for research. Most critically, as is often the case in professional development research, the total sample of teachers (*n* = 7) is small to accommodate the costs of teacher training, observation, and coaching (Schacter, 2015). In turn, this limits the representativeness of the findings and the power of inferential analyses. Although a small sample allows for detailed coding and rich descriptive data, gathering parallel information from a larger array of classrooms would support firm statistical conclusions and, ultimately, generalizability to the Early Head Start population and beyond.

A second concern is that these seven teachers read different books to children, which can foster different kinds of talk. For example, some books were narratives (such as *Baby Loves Winter*), whereas others were more informational or participatory (*Shake a Leg! Elmo*). In our experience, allowing teachers to choose the book they would like to read helps to ensure that observations are representative of what typically happens in a class day (i.e., ecological validity); however, using the same book across all settings facilitates classroom comparisons. Future research might include both approaches and directly compare them. As a related point, more examination of the texts that teachers read to/with toddlers is needed. In particular, we found that some texts mentioned target words more than others, and some included questions (e.g., the refrain, “Is this the bus for us, Gus?”) that could potentially support children’s talk. Careful analyses of the teacher-child discourse around a wider array of texts in a larger sample could be helpful.

A third issue is that it was beyond the scope of this implementation-focused study to include more detailed child data. The most important dimension of any classroom-based program is its role in improving children’s outcomes, ideally explored through rigorous methods that allow for causal conclusions. One future approach would be to include rigorous background information (e.g., exposure to English and other languages at home), as well as standardized and/or project-based measures of children’s skills. Also of value, however, would be tracking individual children during observations to gauge which/how many children offer the most responses, are the most accurate, or even never responds. Individual-level data of this nature would potentially elucidate why teachers ask the specific questions they choose and would also help teachers better tailor their instruction to children’s skills.

## Conclusion

Little is known about the CDS that teachers use to communicate with toddlers in classrooms. This study examined CDS as well as child talk during book readings in American Early Head Start classrooms serving 2-year-olds from households in poverty, as teachers piloted a new, light-touch PD model. Overall, the environment was similar in global quality and linguistic complexity to prior observations of toddler book readings, but close analysis of teacher and child talk strategies revealed potential differences between Askers (more focused on closed questions), Tellers (more focused on comments), and those with a Balanced approach to questions and comments. Overall, this study weaves together a more comprehensive perspective on toddler classroom language, illuminating pathways for future research.

## Data Availability Statement

The dataset presented in this article is not readily available because these videos are protected by Temple University’s IRB. Requests to access the datasets should be directed to AH (annemarie.hindman@temple.edu).

## Ethics Statement

The studies involving human participants were reviewed and approved by the Temple University IRB. The patients/participants provided their written informed consent to participate in this study.

## Author Contributions

AH was the PI of the grant and led the data analysis and manuscript writing. JF conducted the coding and analysis of syntax. KA conducted the coding with the CLASS. BW and PS are project PIs as well, helping to conceptualize the project, and design and implement the teacher professional development. All authors reviewed and approved the manuscript.

## Author Disclaimer

The opinions expressed are those of the authors and do not represent views of the Institute or the U.S. Department of Education.

## Conflict of Interest

The authors declare that the research was conducted in the absence of any commercial or financial relationships that could be construed as a potential conflict of interest.

## Publisher’s Note

All claims expressed in this article are solely those of the authors and do not necessarily represent those of their affiliated organizations, or those of the publisher, the editors and the reviewers. Any product that may be evaluated in this article, or claim that may be made by its manufacturer, is not guaranteed or endorsed by the publisher.
